# More optimal relativistic quantum key distribution

**DOI:** 10.1038/s41598-022-15247-x

**Published:** 2022-09-13

**Authors:** Georgi Bebrov

**Affiliations:** Telecommunications Department, Technical University of Varna, 9010 Varna, Bulgaria

**Keywords:** Quantum information, Information theory and computation

## Abstract

A great challenge in the field of quantum cryptography is the design and implementation of optimal quantum key distribution (QKD) scheme. An optimal scheme in terms of security is the so-called relativistic quantum key distribution; it ensures the security of the system by using both quantum phenomena and relativity. However, the existing relativistic schemes have not demonstrated optimality in terms of efficiency and rate (including secret key rate). Here we report two point-to-point relativistic quantum key distribution schemes implemented with weak coherent pulses. Both schemes rely on high-dimensional quantum systems (phase and polarization encodings are utilized for establishing key bits). One of the proposed schemes is a system comprised of two sequentially connected interferometers, as the first (interferometer) controls the behavior of the second one. The other proposed scheme represents a setup of a classic relativistic QKD, but with slight modification. Both of the proposed schemes are characterized with high secret key rate. The latter scheme has the highest secret key rate of all the relativistic QKD protocols. However, the values for the secret key rate are relevant for distances of up to 150 km. The former scheme has lower secret key rate, but longer operating distances (the work could operate at distances of up to 320 km). Those values of rate are obtained without disturbing the security. Secret-key-rate comparison between distinct models is reported. The proposed relativistic models are compared to twin-field QKD protocols. Furthermore, the work proposes a metric for evaluating the optimality of a QKD. It is defined as a ratio between the secret key rate (at a given distance) and the amount of quantum resources (qubits) used in the QKD of concern. It is shown that one of the proposed schemes in this article is the most optimal relativistic key distribution and more optimal than the original twin-field. It is also verified that the proposed schemes excels the original twin-field in terms of secret key rate, but for short distances.

## Introduction

The quantum key distribution^1–9^ (QKD) is a communication model with information-theoretic security. Its security is provided by the laws of quantum physics. The existing QKD models are mainly divided into two groups: discrete-variable (DV) schemes^1–9^ and continuous-variable (CV) schemes^10–12^. Detailed reviews on these two types of QKD are given in Refs.^13,14^. In this paper, we are concerned with the discrete-variable implementation of the quantum key distribution. Due to practical difficulties in implementing the standard DV QKD protocols, researchers resort to developing novel models and techniques, which are used to improve the existing schemes and mitigate the effects of the practical loopholes. For instance, for overcoming some security issues, the so-called measurement-device-independent or just device-independent schemes^9,15–35^ are developed. A practical issue for the DV QKD protocols is the lack of existing a true single-photon source, which is required for the proper work of the above-mentioned models. For this reason, the well-known decoy-state technique is introduced^36,37^. It is used to implement the existing models with weak coherent pulse (WCP) states instead of single-photons without deteriorating the behavior of the QKD process. In this regard, WCP protocols are developed^6,38–41^. Nowadays, the state of the art is the so-called twin-field QKD, which is initially introduced in Ref.^39^ and later modified in the works of Refs.^40–43^. The twin-field QKD protocols manifest a secure key rate that scales with the square root of the channel transmittance, as stated in Ref.^39^. It represents a practical counterpart of the measurement-device-independent model. The point-relay-point structure of such protocols allows a higher-distance quantum key distribution implementation^9,39^.

The main parameter of the QKD schemes is the secret key rate, which in general is illustrated as a function of the operating distance. It shows the reach and the capacity of a given QKD. So far, the twin-field protocols are characterized with the best secret key rate behavior—they demonstrate a balanced rate-to-distance graph (these models maintain satisfactory rate for longer distances). The secret key rate is actually an expression yielding a rate value when as many as possible (or almost all) negative practical QKD effects are taken into account. In this connection, many works are introduced^44–47^, which involve tight security bounds and finite-key analysis. The latter allows for better modelling the practical realizations of the QKD system.

Optical interference is one of the practical tools for constructing secure quantum key distribution systems between two parties^38,39,48^. The process of interference allows the development of the so-called relativistic quantum key distribution^48–51^. This type of QKD relies mainly on interferometric setup and the principles of relativity^52–54^ in order to provide a way to detect the presence of a third party (eavesdropper). The other quantum phenomena (such as the uncertainty principle) are profitable resources, which could be used in the implementation of the relativistic models. So, they could be used for further improving such schemes. Throughout the years, several relativistic schemes are introduced^48–51^. In Ref.^49^, a single-photon interferometric setup together with delay lines is proposed. The authors state that the usage of orthogonal states are sufficient for ensuring a secure quantum key distribution of this type. The system manifests a rate of one bit per setup use. However, the work of Ref.^49^ is based on single-photon interferometry, it is not practical one. Ref.^50^ presents a single-photon (or WCP) relativistic scheme, which relies upon the random choice of unitary operator (selecting one operator out of two) independently performed by the participants (sender and recipient). Reference^48^ introduces a setup in which two WCP states (signal and reference states) prepared by the sender interfere at the recipient. The sender controls the phase of the signal state whereas the recipient controls the phase of the reference state. This scheme is characterized with a rate of 1 bit per relevant setup use. A relevant setup use implies a transfer and interference processes, which produce a click at the recipient’s detector. The work of Ref.^51^ reports a setup in which two high-dimensional WCP states (both polarization and phase encoding are applied on the WCPs) interfere at the recipient. It is characterized with a rate of 2 bits per relevant setup use. As just mentioned, the implementation of a relativistic scheme requires a distribution of two quantum signals over two distinct paths. This is accompanied with lowering the rate-to-distance behavior and resource efficiency of the communication system. Another drawback of the relativistic schemes is the need of reliable and precise synchronization system, which is fundamental for this kind of key distribution^48^. In order to compensate the complexity of the synchronization system, the relativistic communication link needs to be as practical as possible. Also, in order for the relativistic key distribution to be as secure as possible, its transfer rate acquires relatively low values^48,50^. A way to increase the rate is to incorporate quantum phenomena into the transfer process of the relativistic quantum key distribution^51^.

In this article, we present a relativistic key distribution scheme, which is more optimal than the existing ones in terms of rate as well as efficiency. By rate it is meant not only the communication (or transfer) rate mentioned above (measured in [bits/use]), but also the secure key rate (rate-to-distance behavior). The implementation of this QKD is based on weak coherent pulses (WCP), i.e., it is as practical as possible. Note that the security of the novel QKD is not influenced by introducing improvements in the transfer process.

## Results

For the sake of the paper’s aim, in this section, we propose two interferometric schemes appropriate for relativistic quantum key distribution.

**Scheme I:**

**Table 1:** Encoding/decoding table of Scheme I.

Polarization	PSA ($e^{i\phi _{a}}$)	PSB ($e^{i\phi _{b}}$)	Message
${|{z+}\rangle }$	180-deg ($e^{i\pi }$)	180-deg ($e^{i\pi }$)	00
${|{z-}\rangle }$	180-deg ($e^{i\pi }$)	180-deg ($e^{i\pi }$)	01
${|{x+}\rangle }$	0-deg ($e^{i0}$)	0-deg ($e^{i0}$)	10
${|{x-}\rangle }$	0-deg ($e^{i0}$)	0-deg ($e^{i0}$)	11
${|{z+}\rangle }$	180-deg ($e^{i\pi }$)	0-deg ($e^{i0}$)	10
${|{z-}\rangle }$	180-deg ($e^{i\pi }$)	0-deg ($e^{i0}$)	11
${|{x+}\rangle }$	0-deg ($e^{i0}$)	180-deg ($e^{i\pi }$)	00
${|{x-}\rangle }$	0-deg ($e^{i0}$)	180-deg ($e^{i\pi }$)	01

**Figure 1:**
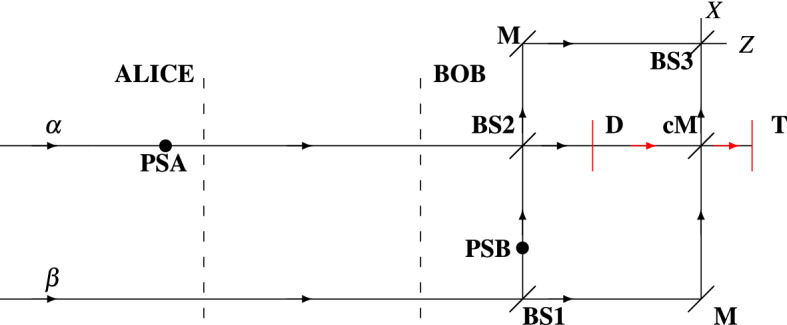
Mach–Zehnder interferometric scheme of the proposed relativistic QKD model. *PSA* phase shift possessed by Alice, *PSB* phase shift possessed by Bob, $\alpha$ signal coherent state, $\beta$ reference coherent state, *BS* beam splitter, *M* mirror, *cM* controlled mirror, *T* terminator, *PSA* phase shift possessed by Alice, *D* detector, *Z* *Z*-basis measurement system, *X* *X*-basis measurement system.

The proposed scheme, which could be regarded as a combination of the setups introduced in Refs.^38,48,51^, is characterized with the illustration in Fig. 1. At the input of the interferometric scheme, two WCP states are fed: $\alpha$ (at time $t_1$) and $\beta$ (at time $t_0$). The state $\beta$ is a reference state and $\alpha$ is a signal state, as $\beta$ is two times “stronger” than $\alpha$ (this is a requirement for the proper operation of the scheme), see Refs.^38,51^. Both WCP states could reside in one of the following polarization states: ${|{z+}\rangle }$, ${|{z-}\rangle }$, ${|{x+}\rangle }$, ${|{x-}\rangle }$. The states ${|{z+}\rangle }$, ${|{z-}\rangle }$ are the eigenstates of the *Z* polarization basis, whereas the states ${|{x+}\rangle }$, ${|{x-}\rangle }$ are the eigenstates of the *X* polarization basis. Note that $\alpha$ and $\beta$ are prepared in identical polarization state. The key bits established by $\alpha$ depends on the polarization state and the phase shifts **PSA** (phase shift of Alice), **PSB** (phase shift of Bob), see Table 1 for reference. As can be seen from the table, Alice sends to Bob a ${|{x\pm }\rangle }$ state only if $\phi _a$ = 0 (**PSA** = 0-deg) and a ${|{z\pm }\rangle }$ state only if $\phi _a$ = $\pi$ (**PSA** = 180-deg).

For the sake of clarity, we describe the way in which Alice and Bob establish correlated key bits. To begin the key distribution, Alice generates weak coherent pulses $\alpha$ (signal state) and $\beta$ (reference state). She at random selects the polarization state in which they will be transferred (one of the polarization states ${|{z+}\rangle }$, ${|{z-}\rangle }$, ${|{x+}\rangle }$, ${|{x-}\rangle }$ ). Alice selects the phase shift **PSA** (**PSA** $\in$ {0-deg,180-deg}), which will be applied to $\alpha$ during its transfer along the interferometric communication scheme. The selection is made according to the following principles: **PSA** = 0-deg if ${|{x\pm }\rangle }$ is prepared; **PSA** = 180-deg if ${|{z\pm }\rangle }$ is prepared. Alice then sends $\beta$ to Bob at time $t_0$ along the lower arm of the interferometric scheme of Fig. 1. At time $t_1$, Alice sends $\alpha$ to Bob along the upper arm of the interferometric scheme of Fig. 1. Slightly after $t_1$, the phase shift **PSA** ($\phi _a$) is applied to $\alpha$. This results in the transformation: $\alpha$ $\rightarrow$ $e^{i\phi _{a}}\alpha$. At the receiving side of the interferometer, $\beta$ is sent through a mainly transmitting beam splitter (**BS1**). A fraction of $\beta$ (denoted by $\beta '$), which is equal to $\alpha$, is reflected off (**BS1**) and forwarded to the beam splitter **BS2**. Then, $\beta '$ is subjected to a phase shift **PSB** (**PSB** $\in$ {0-deg,180-deg}), which is randomly chosen by Bob. The phase state of $\beta '$ is equal to $e^{i\phi _b}e^{i\frac{\pi }{2}}\beta '$, where $e^{i\frac{\pi }{2}}$ = *i* identifies the reflection off **BS1** and $e^{i\phi _b}$ is the phase inherited from **PSB**. In other words, it is situated in the state $ie^{i\phi _b}\beta '$. The other fraction, denoted by $\beta ''$, is forwarded to a mirror, which navigates $\beta ''$ to a controllable mirror (**cM**). The mirror is controlled via a signal produced by a detector system **D** ($\delta$ state should be present at the detector, see Fig. 3): if $\delta$ is present, **cM** gets enabled and $\beta ''$ is directed towards a terminator (**T**); otherwise, **cM** is disabled [*Note*: In this description, we neglect the time delay in generating a signal for the sake of controlling **cM**. If we take into account such a delay, delay lines should be incorporated into $\gamma$ and $\beta ''$ paths; only then the delay is compensated and the proposed scheme is completely compliant with the nature of space-time]. At time $t_2$, the signal state $e^{i\phi _a}\alpha$ and the reference state $ie^{i\phi _b}\beta '$ interfere at **BS2** as follows:

1 $$\begin{aligned} e^{i\phi _{a}}\alpha \circ ie^{i\phi _b}\beta ' = {\left\{ \begin{array}{ll} \delta &{} \text {if }e^{i0}\alpha \circ ie^{i\pi }\beta ',\\ -\delta &{} \text {if }e^{i\pi }\alpha \circ ie^{i0}\beta ',\\ i\gamma &{} \text {if }e^{i0}\alpha \circ ie^{i0}\beta ', \\ -i\gamma &{} \text {if }e^{i\pi }\alpha \circ ie^{i\pi }\beta ', \end{array}\right. } \end{aligned}$$

where “$\circ$” denotes the operation *interference*, $\gamma$ identifies the state characterizing the upper output of **BS2**, and $\delta$ identifies the state characterizing the lower output of **BS2**. If $\phi _a$ and $\phi _b$ are so chosen that $\pm \delta$ occurs after the interference process, a signal is forwarded to the detector system **D**. If $\phi _b$ = 0, **D** is adjusted to the *Z*-basis measurement system, else ($\phi _b$ = $\pi$) **D** is adjusted to play the role of a *X*-basis measurement system. A click is interpreted as a message (a two-bit symbol) according to Table 1. As mentioned in the above lines, the triggered detector of **D** generates a signal, which makes $\beta ''$ to be reflected off the controllable mirror **cM**. Then, the state $\beta ''$ is directed to the terminator **T**. In this scenario, Bob accounts no click at either *X* or *Z* measurement basis of **BS3**, see Fig. 1. If $\phi _a$ and $\phi _b$ are so chosen that $\pm i\gamma$ occurs after the interference process, the controllable mirror **cM** is disabled and $\beta ''$ moves to **BS3**. Note that $\beta ''$ reaches **BS3** as $e^{i\frac{\pi }{2}}\beta ''$, where $e^{i\frac{\pi }{2}}$ is induced from reflection off a mirror **M**. Therefore, the phase state of $\beta ''$ at **BS3** is $i\beta ''$. On the other hand, at the upper output of **BS2** $\pm i\gamma$ gets an additional phase of $e^{i\frac{\pi }{2}}$ by reflecting off a mirror **M**: $\pm ie^{i\frac{\pi }{2}}\gamma$ $\rightarrow$ ${\mp } \gamma$, where $+\gamma$ = $e^{i0}\gamma$ and $-\gamma$ = $e^{i\pi }\gamma$. Then, $\gamma$ is forwarded to **BS3**. At this beam splitter, the following interference occurs:

2 $$\begin{aligned} {\mp }\gamma \circ i\beta '' = {\left\{ \begin{array}{ll} \eta &{} \text {if }e^{i0}\gamma \circ i\beta '',\\ -i\zeta &{} \text {if }e^{i\pi }\gamma \circ i\beta '', \end{array}\right. } \end{aligned}$$

where $\eta$ identifies the state characterizing the upper output of **BS3**, and $\zeta$ identifies the state characterizing the lower output of **BS3**. The upper output of **BS3** is connected to *X*-basis measurement system, while its lower output is connected to *Z*-basis measurement system. The key string of Bob is constructed according to the measurements (detections) taken place and the relations “phase-polarization” introduced in Table 1: a detection corresponds to a given phase and polarization. Therefore, based on any detection (present at **D** or *X*-basis measurement system or *Z*-basis measurement system), Bob extracts two-bit key symbols. We should point out that after obtaining his key Bob announces the outputs of **BS2** at which each signal is detected ($\gamma$ $\rightarrow$ 0; $\delta$ $\rightarrow$ 1). Based on the information announced by Bob, her phase shifts **PSA**, and the polarization states in which distinct $\alpha$s and $\beta$s are prepared, Alice constructs her sifted key. If no errors are present in the communication channel connecting Alice and Bob, they would have totally correlated sifted keys. Since a noiseless channel does not exist in reality, the sifted keys of Alice and Bob differ from each other. In order to be established a completely correlated, secure key between the two parties, they perform parameter estimation, key reconciliation^55^, and privacy amplification^56^. In this relativistic communication between Alice and Bob, both parties are aware of the time at which the communication begins and the time interval between sequential signals $\alpha$ (or $\beta$). This implies that if the spatial measures (e.g., length *L*) of the scheme are preliminary known, Bob (the recipient) is aware of the time instances at which he could detect signal at either **D** or *X* and *Z* measurement systems. Based on this knowledge, Bob determines whether or not a given measurement (detection) is retarded. If a measurement is retarded, it is considered as eavesdropped and its result is discarded. Note that Bob announces the discarded measurements; this is included in the process of correlating the keys of Alice and Bob.

**Scheme II:**

**Figure 2:**
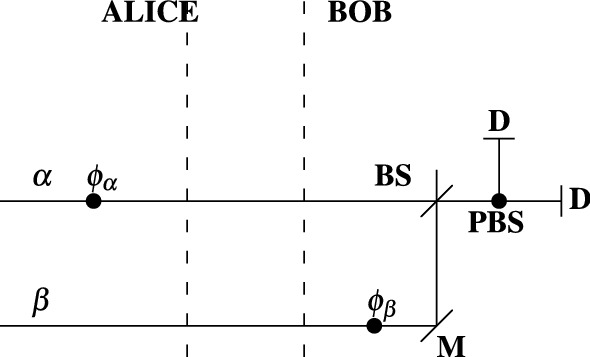
Modified Ref.^48^ relativistic quantum key distribution scheme. *BS* beam splitter, *M* mirror, $\alpha$,$\beta$ weak coherent states ($\mu _{\beta }$ = $\mu _{\alpha }$), *D* detector, *PBS* polarizing beam splitter, $\phi _{\alpha }$,$\phi _{\beta }$ independent phase operators ($\phi _{\alpha }$,$\phi _{\beta }$ $\in$ {0-deg,180-deg}).

This scheme operates as follows. Alice generates two WCP states $\alpha$ and $\beta$ ($\alpha$ = $\beta$). Alice feeds $\alpha$ into the upper arm of the interferometric setup of Fig. 2, whereas $\beta$ is fed into the lower arm. Alice at random selects the phase shift $\phi _{\alpha }$ ($\phi _{\alpha }$ $\in$ {0-deg,180-deg}), which is applied to $\alpha$. Based on the phase shift, Alice at random selects a polarization state for both $\alpha$ and $\beta$: if $\phi _{\alpha }$ = 0-deg, X-basis polarization state (${|{x+}\rangle }$, ${|{x-}\rangle }$) is chosen; if $\phi _{\alpha }$ = 180-deg, Z-basis polarization state (${|{z+}\rangle }$, ${|{z-}\rangle }$) is chosen. For instance, if Alice selects $\phi _{\alpha }$ = 0-deg, then a possible polarization state is ${|{x+}\rangle }$ (diagonal polarization state). The WCP states travel from Alice to Bob. Bob performs a random phase shift $\phi _{\beta }$ on $\beta$ ($\phi _{\beta }$ $\in$ {0-deg,180-deg}). Also, based on $\phi _{\beta }$, Bob adjusts his polarizing beam splitter **PBS**: if $\phi _{\beta }$ = 0-deg, Z-basis polarization measurement is conducted at **PBS**; if $\phi _{\beta }$ = 180-deg, X-basis polarization measurement is conducted at **PBS**. As can be easily verified, in half of the times the interference $e^{i\phi _{\alpha }}\alpha \circ ie^{i\phi _{\beta }}\beta$ at the beam splitter **BS** leads to a click at one of the detectors **D**, see Fig. 2 for reference. The click is considered as a signal for establishing a key symbol. Bob records a two-bit key symbol, which is related to his phase shift $\phi _{\beta }$ and a detector click: $\phi _{\beta }$ = 0-deg, upper detector clicks $\rightarrow$ ‘00’; $\phi _{\beta }$ = 180-deg, upper detector clicks $\rightarrow$ ‘10’; $\phi _{\beta }$ = 0-deg, lower detector clicks $\rightarrow$ ‘01’; $\phi _{\beta }$ = 180-deg, lower detector clicks $\rightarrow$ ‘11’. For the sake of clarity, we present the way of establishing a key symbol in Table 2.

Bob announces the instances at which the detector clicks; the other instances (delayed clicks, inconclusive measurements or no clicks at certain time instances) are discarded (sifted): ‘0’ $\rightarrow$ click (conclusive measurement); ‘1’ $\rightarrow$ no click (inconclusive measurement). Based on this message, Alice records a key symbol according to Table 2: knowing her phase shift $\phi _{\alpha }$ as well as the polarization state of $\alpha$ and $\beta$, Alice learns the phase shift $\phi _{\beta }$ of Bob. That is, Alice obtains a correlated key symbol with Bob.

**Table 2:** Encoding/decoding table of **Scheme II**. We assume that the polarization state ${|{x(z)+}\rangle }$ is reflected off the Bob’s **PBS**, while ${|{x(z)-}\rangle }$ is passed towards the lower detector **D**.

Polarization	$\phi _{\alpha }$	$\phi _{\beta }$	D	Message
${|{x+}\rangle }$	0-deg	180-deg	Upper detector	00
${|{x-}\rangle }$	0-deg	180-deg	Lower detector	01
${|{z+}\rangle }$	180-deg	0-deg	Upper detector	10
${|{z-}\rangle }$	180-deg	0-deg	Lower detector	11

## Discussion

In this section, we present an analysis of the proposed relativistic schemes. The analysis consists of discussing the behaviour of the schemes in terms of security, transfer rate, transfer efficiency, secret key rate, and resource optimality.

The security of the proposed schemes is analyzed with regard to coherent and *intercepting attacks* (e.g., intercept-resend attack or intercept-resend attack with preliminary prepared state). As noted in Ref.^51^, a relativistic scheme, which uses two encodings (phase and polarization encodings), is secure against intercepting attacks if it meets the following requirements: (i) the relativistic quantum key distribution utilizes a two-arm interferometric setup; (ii) the scheme utilizes two or more polarization bases when polarization encoding is utilized. Requirement (i) ensures that the presence of an eavesdropper will be revealed if an ordinary intercept-resend attack or intercept-resend attack with preliminary prepared systems is launched. This is due to the fact that the act of interception leads to distorting the space-time paths of the transferred WCPs. The distortion causes delayed measurement results being taken into account by the participants (sender and recipient) of the scheme. In the Supplementary Material, we give details on the way how an interception attack distorts the space-time path of an intercepted WCP state. Also, we give a proof on that an eavesdropper cannot intercept the transferred states in an unhindered manner. Requirement (ii) ensures that the presence of an eavesdropper will be revealed if an ancilla is appended to the signal state. Note that if one polarization basis is used in the scheme, the eavesdropper will in an unhindered manner append ancilla and gain information about the polarization of the signal state^51,57^. More details on this attack are given in the Supplementary Material.

**Figure 3:**
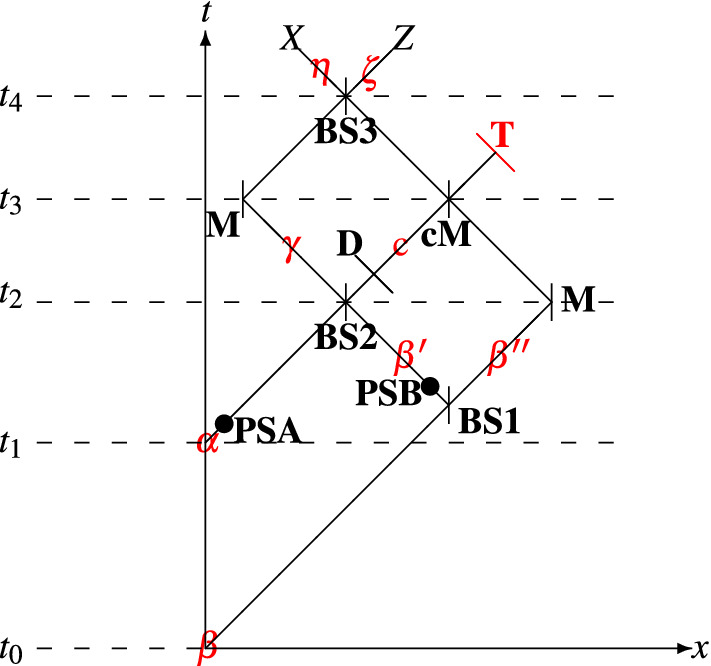
Space-time diagram of a communication scheme proposed for relativistic QKD. $\alpha$ signal (weak) state, $\beta$ reference (strong) state, *PSA* phase shift possessed by Alice, *PSB* phase shift possessed by Bob, *BS* beam splitter, *D* detector, *M* mirror, *cM* controlled mirror, *T* terminator.

In the following lines, we calculate the transfer efficiency and rate of the proposed schemes. The so-called *transfer efficiency of quantum systems* (*weak coherent pulses*) is expressed as

3 $$\begin{aligned} E = \frac{k}{q}, \end{aligned}$$

where *k* is the amount of relevant quantum systems (weak coherent pulses) and *q* is the overall amount of quantum systems used in a relativistic quantum key distribution scheme. By relevant quantum systems we mean those, which are used to establish the so-called sifted key in a quantum key distribution. The *transfer rate*, in [*bits*/*use*], is given by^51^

4 $$\begin{aligned} R = \frac{n}{m}, \end{aligned}$$

where *n* is the size (length) of the sifted key established in a relativistic quantum key distribution and *m* is the number of instances in which the setup (Fig. 1) is used for transferring *n*-bit key. In the following, we determine both *E* and *R* of **Scheme I** and **Scheme II**. As described in **Scheme I**, any transferred quantum system is a relevant system, because each system is used to transfer 2 bits of information, as mentioned in the previous section. [*Note*: In determining *E* we neglect the presence of an eavesdropper so that we do not take into account the measurements (detections), whose space-time features are disturbed (the time of measurement is delayed due to the presence of an eavesdropper)]. This implies that the efficiency of **Scheme I** is $E_{\text {I}}$ = 1. Taking into consideration the information carriage of the transferred weak coherent pulses at each use of the setup, the transfer rate is therefore $R_{\text {I}}$ = $\frac{m\cdot 2}{m}$ = 2 [*bits*/*use*]. In **Scheme II**, half of the transferred systems are sifted. Also, the non-sifted systems are used to establish 2 bits of information (two non-sifted systems are involved in establishing a two-bit key symbol). This implies that the efficiency of **Scheme II** is $E_{\text {II}}$ = 0.5. The communication (transfer) rate of **Scheme II** is $R_{\text {II}}$ = $\frac{\frac{m}{2}\cdot 2}{m}$ = 1 [*bit*/*use*].

In order to show the novelty of the work presented in this article, we compare the proposed relativistic schemes to existing ones^48,50,51^. The comparison is carried out in terms of transfer rate and efficiency. To show the comparison between the proposed and existing relativistic schemes, we present Table 3 in which their rates and efficiencies are collated. Herein, we omit calculating the efficiencies and rates of Refs.^48,50,51^. They can be concluded from the analysis introduced in Ref.^51^. Note that a scheme has a value of efficiency *E* = 0.5 because of discarding quantum systems (weak pulses) in the sifting procedure. As can be seen from the table, the proposed relativistic schemes have the highest transfer rates and efficiencies. **Scheme I** excels **Scheme II** in terms of these quantities.

**Table 3:** Comparison between proposed and existing^48,50,51^ relativistic QKD protocols in terms of rate and efficiency. For detailed rate and efficiency analyses of Refs.^48,50,51^, see Ref.^51^.

${{Protocol}}$	Rate	Efficiency
Ref.^48^	0.5	0.5
Ref.^50^	0.5	0.5
Ref.^51^	1	0.5
**Scheme I**	2	1
**Scheme II**	1	0.5

### Secret key rate

We evaluate the proposed relativistic schemes in terms of the following secret-key-rate evaluations^39^

5 $$\begin{aligned} S_{\text {I}} = q\{\underline{Q}_1 |_{\mu ,L}[I - h(\overline{e}_1|_{\mu ,L})]-fQ_{\mu ,L}h(E_{\mu ,L})\}, \end{aligned}$$

which is used for **Scheme I**, and^58–60^

6 $$\begin{aligned} S_{\text {II}} = q[I - h(\text {QBER}) - fh(\text {QBER})], \end{aligned}$$

which is used for **Scheme II**. In these expressions, *h*(.) is the binary Shannon entropy, *q* is the sifting parameter^37^, *I* is the information carriage, and *f* is the efficiency of the error correction algorithm. The information carriage represents the amount of bits transferred by one use of the QKD setup, i.e., it coincides with *R* of Eq. 4. More details on these expressions are given in the Supplementary Material.

**Figure 4:**
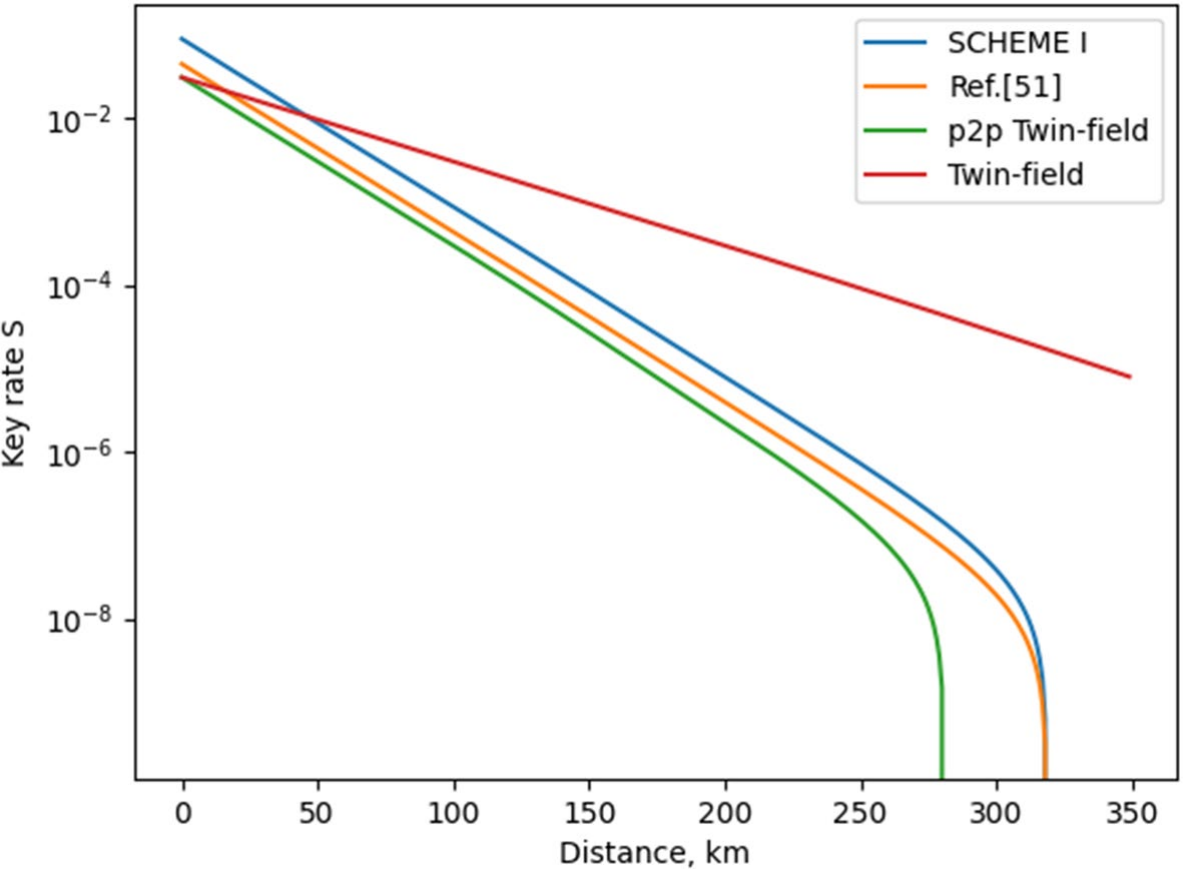
Key rates of different relativistic schemes. Details on the parameters used to evaluate the rates of the distinct schemes are given in the Supplementary Material. We should note that for the proposed scheme, as well as for the work of Ref.^51^, the following relation between $\mu _{\alpha }$ and $\mu _{\beta }$ is used: 2$\mu _{\alpha }$ = $\mu _{\beta }$. Also, $\mu$ in Eq. (5) is defined as $\mu$ = $\mu _{\alpha }$ + $\mu _{\beta }$, as proposed in Ref.^39^. The “p2p Twin field” is a twin field protocol conducted only between two parties; no relay node is used, as illustrated in Fig. 2b of Ref.^39^. The “Twin-field” presents the original model of Ref.^39^. Note that we omit the so-called slice sifting in the rate calculation of the twin-field QKD schemes presented in the figure.

We choose to evaluate **Scheme I** with Eq. (5) because it is used to characterize an identical scheme (twin-field QKD). **Scheme I** resembles Ref.^39^ in encoding (phase encoding) and setup [both outputs of the interferometric beam splitter **BS2** (see Fig. 1 for reference) are used in establishing key bits]. In Fig. 4, we present a comparison between distinct relativistic and twin-field QKD schemes. We suppose that the relativistic models use decoy-state approach. As can be seen from Fig. 4, the proposed scheme displays the best key rate graph from the presented relativistic models. It also excels the point-to-point twin field protocol in terms of rate and distance. We should emphasize on that **Scheme I** is even better than the original twin-field QKD, but only for distances up to around 50km, as can be easily verified in Fig. 4. A problem of the work presented in this paper is the fact that two encodings (phase and polarization encoding) are utilized. As can be seen in the Supplementary Material (Eq. 8), the usage of two encodings leads to higher error rates. For this reason, it is of utmost importance to use a QKD with one encoding. The higher the error rate, the lower the key rate and operating distance. Also, the higher the intrinsic (not caused by an eavesdropper) error rate, the lower the security threshold. In this regard, in future work, we would pay attention to introduce a relativistic scheme relying only on one encoding and having the same rate behavior as the scheme presented in the current paper.

**Figure 5:**
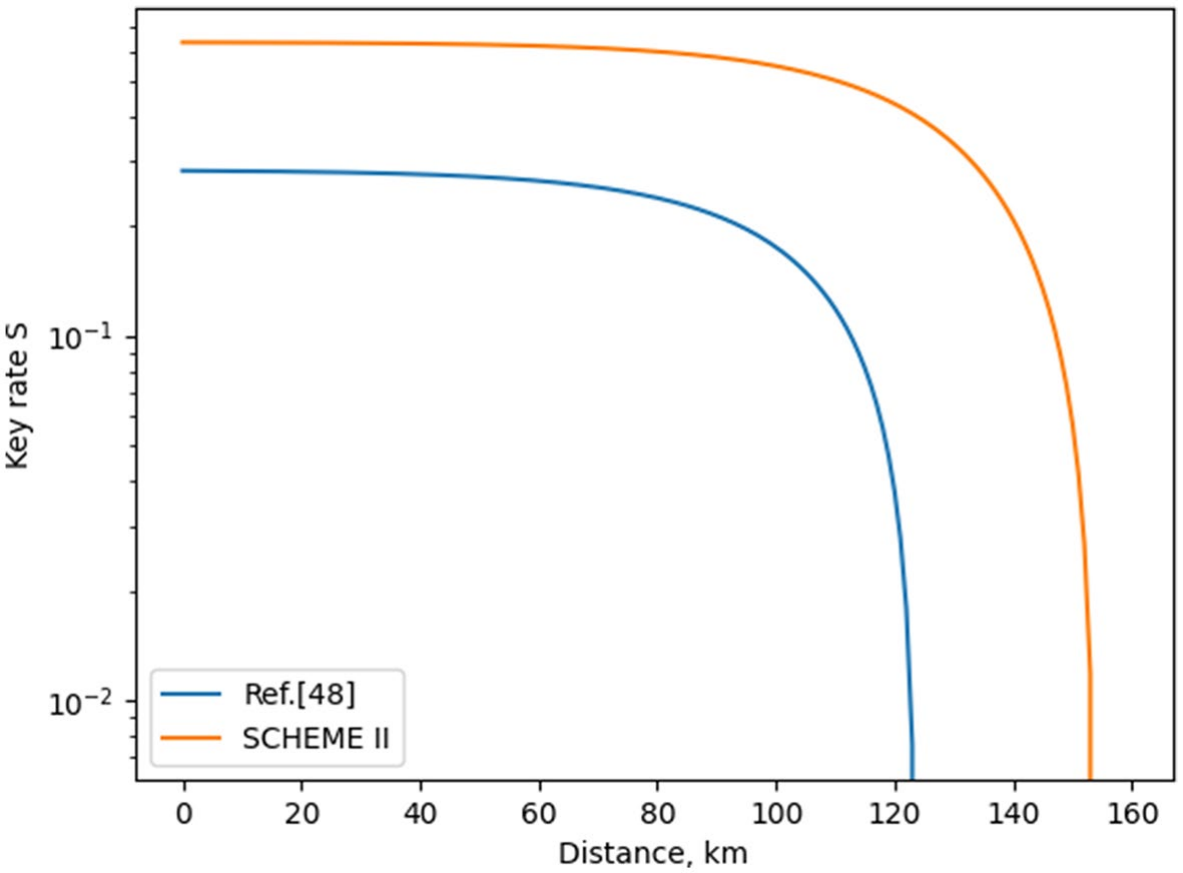
Key rates of classic relativistic schemes. Details on the parameters used to evaluate the rates of the distinct schemes are given in the Supplementary Material.

We choose to evaluate **Scheme II** with Eq. (6) because it resembles the work of Ref.^48^, where identical setup is used and no decoy-state approach is applied. The only difference between **Scheme II** and Ref.^48^ is that two parameters are used for encoding data into quantum systems in **Scheme II**: phase and polarization are employed. In Fig. 5, we present a comparison between **Scheme II** and the work of Ref.^48^.

A way to assess the optimality of a QKD is to use the secure key rate (or the way of using quantum resources) in the following ratio:

7 $$\begin{aligned} \frac{S(d)}{Q} = F(d), \end{aligned}$$

where *S*(*d*) is the secure key rate of the scheme at a given distance *d* and *Q* is the number of quantum WCPs transferred per setup use. As pointed out in the above expression, this evaluation of the optimality is a function of the operating distance. Based on this parameter, we compare **Scheme I** and **Scheme II** to other QKD models. In Table 4, we compare **Scheme I** to the relativistic model of Ref.^51^ and twin-field model of Ref.^39^. In Table 5, we compare **Scheme II** to the relativistic model of Ref.^48^. As can be readily verified by Table 4, **Scheme I** is the most optimal one in terms of secure key rate for lower distances if it could be implemented with only one encoding mechanism, as mentioned above. Actually, as shown in the third column of Table 4, **Scheme I** is almost as optimal as the original twin-field QKD and more optimal than Ref.^51^. In Table 5, we verify that the proposed scheme (**Scheme II**) is more optimal than the work of Ref.^48^. We can therefore conclude that the proposed relativistic schemes of this paper excels in terms of rate and optimality the setups of the existing relativistic QKD protocols^48,51^. Also, it is evident from Tables 4 and 5 that **Scheme II** is the most optimal QKD of those involved in this analysis: **Scheme II** has the highest value of *F*(*d*) for the examined *d* (*d* = 10 km). As can be verified by Figs. 4 and 5, **Scheme II** will be more optimal than the other QKD models for distance of up to 150 km (this is the distance limit of **Scheme II**). So, for short distance, one should prefer using **Scheme II** for the case of relativistic QKD. However, if one needs a QKD system for longer distances, **Scheme I** is to be employed.

**Table 4:** Comparison between **Scheme I** and existing QKD protocols in terms of optimality *F*(*d*) for *d* = 10 km. When high-dimensionality (usage of two encodings) is accounted, the amount *Q* (Eq. 7) is doubled.

Protocol	Optimality	Optimality (high-dimensionality accounted)
Ref.^39^ (point-to-point)	$\frac{0.019}{2}$ = 0.00965	0.00965
Ref.^39^ (original)	$\frac{0.024}{2}$ = 0.012	0.012
Ref.^51^	$\frac{0.028}{3}$ = 0.0093	$\frac{0.028}{6}$ = 0.0047
**Scheme I**	$\frac{0.056}{3}$ = 0.019	$\frac{0.056}{6}$ = 0.0093

**Table 5:** Comparison between **Scheme II** and existing relativistic QKD protocol^48^ in terms of optimality *F*(*d*) for *d* = 10 km. When high-dimensionality (usage of two encodings) is accounted, the amount *Q* (Eq. 7) is doubled.

Protocol	Optimality	Optimality (high-dimensionality accounted)
Ref.^48^	$\frac{0.28}{2}$ = 0.14	0.14
**Scheme II**	$\frac{0.635}{2}$ = 0.3175	$\frac{0.635}{4}$ = 0.15875

## Summary

In summary, we propose two relativistic quantum key distribution schemes (**Scheme I** and **Scheme II**), which are more optimal in terms of secure key rate, transfer rate and efficiency than the existing ones^48,50,51^. This is achieved by using modified interferometric setups. **Scheme I** consists of two sequentially connected interferometers, as the first one controls the operation of the second one. **Scheme II** is identical to Ref.^48^ with the difference that both phase and polarization are used for establishing key symbols in the work of this paper. As a result, we obtain transfer rate (information carriage) of 2 [bits/relevant use] for **Scheme I** and **Scheme II**, which is the highest value achieved so far. However, **Scheme II** is characterized with a sifting process, which implies that the actual (averaged) rate is 1 [bit/use]. In Fig. 4, we present the secret-key-rate graphs of the distinct relativistic QKD models (**Scheme I** is included in the figure). Moreover, the graphs of twin-field QKD models^39^ are depicted; this is done for the sake of finding out the position of the relativistic schemes compared to the state-of-the-art QKD setup, namely, the twin-field model. As seen from Fig. 4, the **Scheme I** excels the twin-field approach for distances up to 50 km. In Fig. 5, we present the secret-key-rate graphs of relativistic protocols, which do not rely on decoy-state approach (**Scheme II** is included in the figure). It is shown that the proposed relativistic model (**Scheme II**) has higher secret key rate as well as operating distance than the work of Ref.^48^. In Tables 4 and 5, we introduce a comparison between distinct models in terms of a function *F*(*d*), which could be used to represent a way of assessing the optimality of QKD protocols. As shown, **Scheme II** excels in terms of *F*(*d*) its relativistic counterparts^48,51^ as well as existing twin-field models^39^ for operating distances of up to 150 km. The improvement in efficiency and rate does not influence in a negative way the security of the proposed scheme. Also, the implementation setups proposed for the novel relativistic quantum key distributions are practical—they are based on generating, transferring, and processing weak coherent pulses. We should point out that the current work achieves the above-mentioned results at the cost of reducing its practicality. This is related to the fact that two encoding mechanisms (phase encoding and polarization encoding) are involved in the proposed schemes of the current work. The use of two encoding mechanisms increases the error rate, which in turn decreases the secret key rate, the operating distance, and the optimality, as shown in Tables 4 and 5.

## Supplementary Information

Supplementary Information.

## Data Availability

All data generated or analysed during this study are included in this published article. Further details concerning the current study are available in the Supplementary Material accompanying this paper.
