# Supplementary material for: More optimal relativistic quantum key distribution
Source: Sci Rep. 2022 Sep 13;12:15377. doi: 10.1038/s41598-022-15247-x (PMC9470693; doi:10.1038/s41598-022-15247-x)
Supplement: Supplementary file 1 — Supplementary Information. [file 41598_2022_15247_MOESM1_ESM.pdf]

Supplementary Material
"More Optimal Relativistic Quantum Key Distribution"

ABSTRACT

Note: In the following lines, we use reference numbering (which is different from the manuscript) appended at the end of the supplementary material.

1 Secure key rate calculation

The following equations, presented in the Supplementary Material of Ref.[1], are used to calculate the rate of **SCHEME I**. Here is Equation 5 of the manuscript

$$S_{\rm I} = q\{\underline{Q}_1|_{\mu,L}[I - h(\overline{e}_1|_{\mu,L})] - fQ_{\mu,L}h(E_{\mu,L})\}, \tag{1}$$

where $h(.)$ is the binary Shannon entropy, q is the sifting parameter, I is the information carriage, and f is the efficiency of the error correction algorithm. The information carriage represents the amount of bits transferred by one use of the QKD setup. In order to evaluate the parameters (coefficients) involved in this expression, we utilize the following:
Single-photon yield:

$$\underline{y}_1 = [u^2 Q_v e^v - u^2 Q_w e^w - (v^2 - w^2)(Q_u e^u - \underline{y}_0)]/[u(uv - uw - v^2 + w^2)], \tag{2}$$

Single-photon error:

$$\overline{e}_1 = (E_v Q_v e^v - E_w Q_w e^w)/[(v-w)\underline{y}_1], \tag{3}$$

Background yield:

$$\underline{y}_0 = (vQ_w e^w - wQ_v e^v)/(v-w), \tag{4}$$

u-state gain

$$Q_u = 1 - (1 - P_{dc})^2 e^{-u\tilde{\eta}}, \tag{5}$$

Overall transmittance:

$$\tilde{\eta} = \eta_{det} \times \eta, \tag{6}$$

Channel transmittance:

$$\eta = 10^{-\alpha L/10}, \tag{7}$$

Overall QBER for u-state:

$$E_u = \frac{1}{2} + \frac{1}{(2Q_u)(1-P_{dc})}[e^{-u\tilde{\eta}[1-(e_{opt}+e_{pol})]} - e^{-u\tilde{\eta}[e_{opt}+e_{pol}]}], \tag{8}$$

Single-photon gain:

$$\underline{Q}_1|_{\mu,L} = ue^{-u}\underline{y}_1, \tag{9}$$

All states gain:

$$Q_{\mu,L} = p_u Q_u + p_v Q_v + p_w Q_w, \tag{10}$$

Overall QBER:

$$E_{\mu,L} = p_u E_u + p_v E_v + p_w E_w, \tag{11}$$

The following initial parameters (values) are used:
Utilized weak coherent states:

$$\mu_{\alpha,\beta} \in \{w/2, v/2, u/2\} \ (\mu \in \{w, v, u\}), \tag{12}$$

where $w = 10^{-4}$, $v = 10^{-2}$, $u = 0.116 \cdot 2$. The state u is so chosen that we could involve the work of Ref.[3] in the rate comparison. The state u represents μ in Equation (5) of the manuscript. It is defined as $u = \mu = \mu\alpha + \mu\beta$, as mentioned in the paper.
Probabilities of the utilized states:

$$p_v = p_w << p_u = 1 - p_v - p_w \lesssim 1, \tag{13}$$

where we select $p_v = p_w = 0.05$ for the twin-field QKD schemes. The infinitely small values of p_v and p_w implies that decoy-state approach is not applied to the QKD. Another initial parameters: $\alpha = 0.2$ dB/km, $P_{dc} = 10^{-8}$, $f = 1.15$, $\eta_{det} = 0.3$, $e_{opt} = 0.03$, $e_{pol} = 0.03$. These values coincide with those used in Ref.[1] (*exception*: the probabilities are chosen independently of Ref.[1]). We add the parameter e_{pol} in order to account the misalignment and instability in the optical systems due to polarization drifts. For the sake of symmetry, we choose e_{pol} to be equal to e_{opt}, i.e., the imperfections of the polarization encoding induce the same amount of errors as the imperfections of the phase encoding.

Note that the overall QBER for w and v states are calculated in an analogous way as E_u[1]. The same holds for the gains Q_w and Q_v—they are calculated with the same equation as Q_u[1].

For evaluating the secret key rate of **SCHEME II**, we use (Equantion 6 of the manuscript)

$$S_{\text{II}} = q[I - h(\text{QBER}) - f h(\text{QBER})]. \tag{14}$$

Here the QBER is calculated with[2]

$$QBER = QBER_{det} + QBER_{opt} = (\frac{p_{noise}}{p_{phot}}) + \pi_{opt}, \tag{15}$$

where $p_{phot} = \mu\eta_{det}\eta$. We assume that p_{noise} (probability to register a count arising from noise) is equal to the probability of dark count ($p_{noise} = P_{dc}$). The parameter π_{opt} is the probability for a photon to wrongly reach a detector. This is equivalent to the parameter e_{opt} considered above. The parameters η_{det} and η coincide with those presented in the above lines. For **SCHEME II**, we modify this expression as follows

$$QBER = (\frac{P_{dc}}{2\mu\eta_{det}\eta}) + e_{opt} + e_{pol}, \tag{16}$$

where the coefficient 2μ comes from the fact that two WCPs are used to establish a key symbol in **SCHEME II**, as pointed out and utilized in Ref.[1]. Also, an addition parameter (e_{pol}) is inserted, as in the case of **SCHEME I**. Note that e_{opt} accounts for the errors arising from phase mismatch between Alice and Bob, while e_{pol} accounts for the errors arising from polarization mismatch between Alice and Bob. In this paper, we assume that $e_{opt} = e_{pol}$. For the evaluation of the secret key rate of **SCHEME II**, we utilize the following values of the initial parameters: $\mu = 0.116$, $\alpha = 0.2$ dB/km, $P_{dc} = 10^{-5}$, $f = 1.22$, $\eta_{det} = 0.2$, $e_{opt} = 0.03$, $e_{pol} = 0.03$. Note that the same values are used in evaluating the work of Ref.[3] to which the proposed scheme **SCHEME II** is compared.

2 Security

In the current section, we present a security analysis in terms of intercepting and coherent attacks. This analysis holds for both **SCHEME I** and **SCHEME II**.

First, we show the way in which the parties (Alice and Bob) detects the presence of an eavesdropper (Eve) when intercepting attack is launched. The attack and its consequences are illustrated in Figure S1. As shown in the figure, Eve uses the same setup as Bob in order to intercept the states sent by Alice. The communication process between Alice, Eve, and Bob is divided into some time intervals: T_1-T_0, T_2-T_1, T_3-T_2, T_4-T_3. We assume that the states transferred by Alice travel always at the speed of light c. In the figure, we analyze only the path of the reference state β (the lower arm of the interferometer shared between Alice and Bob. With green color, we show the actual path of the state β when no interception is present. With red color, we depict the path of the state β when Eve performs an intercepting attack. As can be seen from the picture, the two paths (actual and intercepted) coincide in the first time interval: $L_1 = c(T_1 - T_0) = L'_1 = c(T_1 - T_0)$, where L_i represents the distance travelled by β in the i-th time interval of the actual path and L'_i represents the distance travelled by β in the i-th time interval of the intercepted path. In the second time interval, the consequence of launching the intercepting attack appears, i.e., the path of β is

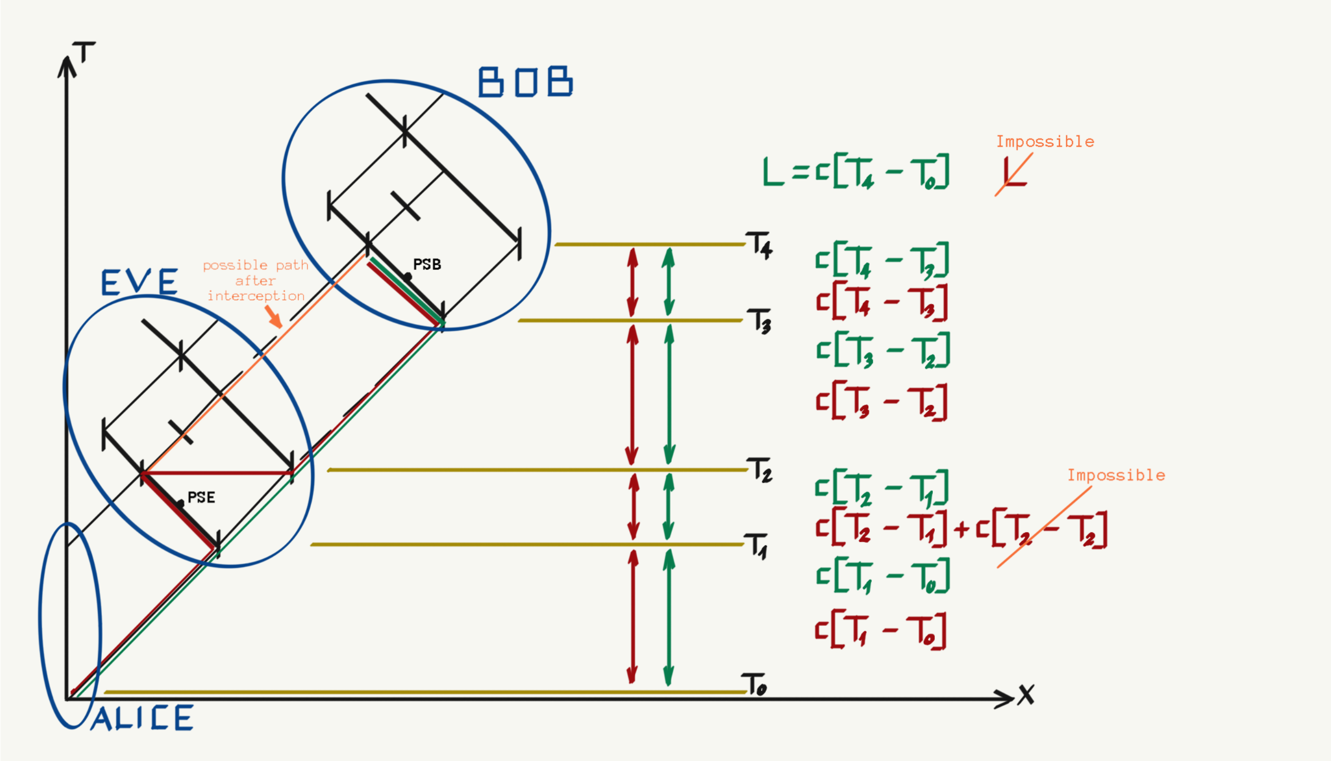

Figure S1. Analysis of an intercepting attack

distorted, as can be easily seen. In order for β to be returned back in its natural path, Eve needs to displace instantaneously β from one location to another. This is characterized by the term $c(T_2 - T_2)$ shown in the picture. However, this is impossible in nature; such a displacement represents a teleportation of a system (information) from one point to another (an action prohibited by the principles of the theory of relativity). That is why, the red path in the picture is considered as impossible. A possible path that the intercepted β could take is the one colored in orange, see Figure S1 for reference. However, the orange path of β coincide in space with the actual path of the signal state α. This implies that α and β would enter Bob's beam splitter at the same input. Then, the interference of those states would not be performed in a way required by the proposed QKD scheme itself. Such an interference would lead to errors. Note that we do not take into account the fact that when intercepted α and β interfere at Eve's side, where a single state (result of the interference) is measured. That is, intercepting the states, Eve always distorts their paths and reveals herself. Bob finds out that such an attack is launched by observing the instant at which a click (a measurement) occurs at his side (*Note*: Bob preliminary knows the time at which Alice sends her states). An option for Eve is to preliminary prepare her own α_e and β_e in a random manner. At the instant of intercepting the states of Alice, then, Eve sends her states to Bob. However, this strategy does not work as well. Preparing α_e and β_e in a way independent of the states of Alice would lead to key mismatch between Alice and Bob. Such a mismatch is detectable during the parameter estimation phase of a QKD protocol. In this way, we show that intercepting attacks in the context of relativistic QKD are detectable and not effective.

We now present the analysis in terms of a coherent attack. The coherent attack consists of attaching an ancilla to the signal state transferred by Alice. In order for the ancilla to copy the signal state, Eve performs a CNOT gate, where the ancilla is the target and the signal state is the control[4]. Mathematically, this is given by

$$|\alpha\rangle |a\rangle \rightarrow |\alpha\rangle |z+\rangle \xrightarrow{\text{CNOT}} \begin{cases} |z+\rangle |z+\rangle, \\ |z-\rangle |z-\rangle, \end{cases} \tag{17}$$

where we choose the signal state α to reside in a Z-basis polarization state. As shown in the above expression, Eve needs to prepare her ancilla in a certain state in order for the signal state to be copied correctly and in an unhindered manner. Suppose now that α resides in a X-basis polarization state:

$$|\alpha\rangle |a\rangle \rightarrow |\alpha\rangle |z+\rangle \xrightarrow{\text{CNOT}} \begin{cases} |x+\rangle_\alpha |z+\rangle_a = (|z+\rangle_\alpha |z+\rangle_a + |z-\rangle_\alpha |z-\rangle_a)/\sqrt{2}, \\ |x-\rangle_\alpha |z-\rangle_a = (|z+\rangle_\alpha |z+\rangle_a - |z-\rangle_\alpha |z-\rangle_a)/\sqrt{2}. \end{cases} \tag{18}$$

This means that the initial α state is converted into a superposition of the Z-basis states. Afterwards, when the α state involves in a process (e.g., interference or measurement) requiring the X polarization basis, the action of appending an ancilla (Equation 18) in the wrong basis leads to the following

$$\begin{cases} |x+\rangle_\alpha |z+\rangle_a = (|z+\rangle_\alpha |z+\rangle_a + |z-\rangle_\alpha |z-\rangle_a)/\sqrt{2} \rightarrow [\frac{1}{\sqrt{2}}(|x+\rangle_\alpha + |x-\rangle_\alpha)|z+\rangle_a + \frac{1}{\sqrt{2}}(|x+\rangle_\alpha - |x-\rangle_\alpha)|z-\rangle_a]/\sqrt{2}, \\ |x-\rangle_\alpha |z-\rangle_a = (|z+\rangle_\alpha |z+\rangle_a - |z-\rangle_\alpha |z-\rangle_a)/\sqrt{2} \rightarrow [\frac{1}{\sqrt{2}}(|x+\rangle_\alpha + |x-\rangle_\alpha)|z+\rangle_a - \frac{1}{\sqrt{2}}(|x+\rangle_\alpha - |x-\rangle_\alpha)|z-\rangle_a]/\sqrt{2}. \end{cases} \tag{19}$$

As can be seen from the last expression, the initial state of α could be flipped with a probability of 1/2 (there is probability of 1/2 for α to reside in its original state ($|x+\rangle_\alpha$ or $|x-\rangle_\alpha$) and there is probability of 1/2 for α to reside in a state opposite to the original one). In a consequent measurement or interference, the superpositional state of α could lead to a wrong result out of these processes (measurement or interference). In this way, during the parameter estimation phase of a QKD protocol, Alice and Bob could reveal the presence of an eavesdropper (could find out that a coherent attack is launched). The above analysis holds also for the case when α is prepared in Z-basis state and the ancilla is prepared in X-basis state.

References

1. M. Lucamarini, Z. Yuan, J. Dynes, and A. Shields, Overcoming the rate–distance limit of quantum key distribution without quantum repeaters, Nature **557**, 400–403 (2018).

2. G. Ribordy, J.-D. Gautier, N. Gisin, O. Guinnard, and H. Zbinden, Fast and User-friendly Quantum Key Distribution, Journal of Modern Optics **47**, 517-531 (1999).

3. K. Kravtsov et al., Relativisitc quantum key distribution system with one-way quantum communication, Scientific Reports **8**, 6102 (2018).

4. F. Yan and X. Zhang, A scheme for secure direct communication using EPR pairs and teleportation, Eur. Phys. J. B **41**, 75 (2004).
